# Combination of Static Magnetic Fields and Peripheral Nerve Stimulation Can Alter Focal Cortical Excitability

**DOI:** 10.3389/fnhum.2016.00598

**Published:** 2016-11-25

**Authors:** Ippei Nojima, Satoko Koganemaru, Tatsuya Mima

**Affiliations:** ^1^Department of Physical Therapy, Nagoya University Graduate School of MedicineNagoya, Japan; ^2^Human Brain Research Center, Kyoto University Graduate School of MedicineKyoto, Japan; ^3^Graduate School of Core Ethics and Frontier Sciences, Ritsumeikan UniversityKyoto, Japan

**Keywords:** transcranial static magnetic stimulation, peripheral nerve stimulation, transcranial magnetic stimulation, motor evoked potential

## Abstract

For clinical application of transcranial static magnetic stimulation (tSMS), it is important to achieve a focal target cortical stimulation. Previous study suggested that the associative stimulation combining non-invasive stimulation of the motor cortex (M1) and the peripheral nerve stimulation (PNS) may be useful to produce cortical excitability change. To test this hypothesis, we measured the M1 excitability and intracortical circuits by using transcranial magnetic stimulation (TMS) before and after the tSMS of short duration (5 min) combined with PNS. Thirty-three normal volunteers were participated; tSMS+PNS (*n* = 11), sham+PNS (*n* = 11), and tSMS alone (*n* = 11). We found the transient suppression of the motor-evoked potential (MEP) of the right abductor pollicis brevis (APB) muscle, but not of the abductor digiti minimi (ADM) muscle, when combining tSMS with PNS over median nerve at the wrist. The lack of suppressive effect on APB in tSMS alone with short duration is in accord with the previous observation. In addition, the tendency of transient enhancement of the short-latency intracortical inhibition was observed immediately after intervention in the tSMS±PNS group. These findings show that the combination of tSMS and PNS can induce the cortical excitability change in target cortical motor area and potentiate the suppression effect.

## Introduction

Noninvasive brain stimulation (NIBS) techniques have become an emerging field in clinical neuroscience due to its effect to modulate cortical excitability (Nitsche and Paulus, 2000, 2001; Reis et al., 2008b) and cognitive or motor function (Iyer et al., 2005; Hummel and Cohen, 2006; Reis et al., 2008a). Among NIBS techniques, transcranial magnetic stimulation (TMS) is widely used for brain stimulation, which can be applied for rehabilitation or therapy for neuropsychiatric disorders (Lam et al., 2008; Koganemaru et al., 2010; Rossini et al., 2010; Dayan et al., 2013; Schulz et al., 2013). Recently, transcranial direct current stimulation (tDCS) is also widely applied in clinical fields because this is a safe, well-tolerated method, which has been shown to induce prolonged excitability changes in humans' cortical regions, resulting in long-term potential (LTP)/depression (LTD)-like synaptic modifications, a cellular correlate of learning and memory (Liebetanz et al., 2002; Nitsche et al., 2003). tDCS is thought to achieve its effects by polarizing neurons and indirectly influencing their firing rates and excitability. Anodal stimulation depolarizes the cell bodies and axon hillock region of corticospinal neurons and increases their excitability, whereas cathodal tDCS has the opposite effect (Nitsche and Paulus, 2000).

Moreover, recent studies reported that local transcranial static magnetic stimulation (tSMS) over the human M1 produced by a small high-powered neodymium magnet can transiently reduce the cortical excitability (Oliviero et al., 2011; Silbert et al., 2013). And we found that the reduction of M1 excitability by tSMS is partly related to the modulation of the short interval intracortical inhibitory circuit (Nojima et al., 2015). Although the physiological mechanisms that underlie this change are not yet known for certain, animal experiments indicated the alteration of the ion channel function embedded in the membrane (Rosen, 2003b). It is possible that high-powered tSMS can affect the orientation of the membrane phospholipids due to their diamagnetic anisotropy. The effect of tSMS was also showed to reduce cortical excitability both in the sensorimotor cortex of humans (Oliviero et al., 2011; Silbert et al., 2013; Kirimoto et al., 2014; Nojima et al., 2015), and in the visual cortex of cats and monkeys (Aguila et al., 2016). Furthermore, the significant behavioral changes were recently reported with the application of tSMS to the visual cortex in monkeys and humans (Gonzalez-Rosa et al., 2015; Aguila et al., 2016).

TMS-based techniques and tDCS deliver electric currents to the cortex to obtain short or long term effects on cortical excitability. The tSMS might be the only NIBS technique that is able to produce a lasting change in cortical excitability that is not associated directly with induced electric currents (Oliviero et al., 2015). And, the advantages of tSMS were its ease of use, absence of an uncomfortable sensation, lack of the need for high operational skill and expensive devices.

However, tSMS has a disadvantage that it is hard to stimulate to the focal target area within cortex. On the other hand, TMS paired with low-frequency peripheral nerve stimulation (PNS) can induce a long lasting, reversible, and somatotopically focal alteration in the human cortical excitability (paired associative stimulation: PAS), which may be related to the associative LTP/LTD (Stefan et al., 2000). Moreover, it was reported that the combination of DCS with low-frequency stimulation in mouse M1 slice also resulted in long-lasting increases in the synaptic efficacy (Fritsch et al., 2010). Here, we hypothesized that tSMS combining with PNS can enhance the plastic change in the target cortical motor area.

## Materials and methods

### Subjects

Thirty-three neurologically healthy subjects (19 males and 14 females; age, 23.6 ± 4.2 years, mean ± SD) participated in this study. None of the participants had a history of neurological or psychiatric disorders by self-report and was under drug treatment during experiment. All subjects were right handed as determined by Oldfield's handedness inventory (Oldfield, 1971). The protocol was approved by the Ethics Committee of Kyoto University Graduate School of Medicine (Kyoto, Japan) and Nagoya University Graduate School of Medicine (Nagoya, Japan). Written informed consent was obtained from all subjects prior to this experiment.

### tSMS exposure

The device we used in this experiment was a cylindrical nickel-plated NdFeB magnet of 50-mm diameter and 30-mm thickness, with a weight of 442 g (Model N-50; NeoMag, Chiba, Japan). The maximum energy density was 406 kJ/m3 (48–51 MGOe), with a nominal strength of 863 N (88 kg). The surface magnetic flux density was about 5340 G. At 2–3 cm from the magnet surface, magnetic field strength of this magnet on the cylinder axis is 120–200 mT (Rivadulla et al., 2014, Kirimoto et al., 2016). A nonmagnetic stainless-steel cylinder, of the same size, weight and appearance as the real magnet, was used for sham stimulation in the control group. The magnet and nonmagnet were positioned by using an arm-type light stand (C-stand, Avenger, Cassola, Italy) over the representational area for the right abductor pollicis brevis (APB) muscle in left M1 identified by TMS and held tangentially against the subject's head. Because it has been reported that the magnetic polarity is irrelevant for neuromodulation (Oliviero et al., 2011), the magnetic field polarity was set north pole oriented toward the subjects.

### Peripheral nerve stimulation (PNS)

The peripheral stimulation consisted of electrical pulses which were 0.2 ms in duration and delivered at rates of 1 Hz through Ag/AgCl conductive adhesive skin electrodes. These electrodes were placed over the right median nerve on the skin 2–3 cm proximal to the distal crease of the wrist with the anode proximal. Stimuli were delivered at the motor threshold for each subject, which is defined as the lowest possible intensity at which a visible muscle contraction of the APB is elicited.

### TMS measurement

TMS was performed with two Magstim 200 magnetic stimulators connected by a bistim module. This device allows delivery of two magnetic stimulations through the same coil. The handle of the coil pointed backwards and 45 degree lateral to the midline. A single pulse of TMS was delivered using a flat figure-of-eight magnetic coil (outer diameter of each wing, 9 cm) at the optimal scalp positions in left M1 to induce a motor response for the right APB. The optimal position was marked on the scalp by a soft-tip pen. The electromyogram (EMG) was recorded from the right APB and Abductor digiti minimi (ADM) using surface silver/silver chloride (Ag/AgCL) electrodes. The reference electrode was placed on tendon, while recording electrode was placed on belly of the muscle. The EMG signals were amplified, band-pass-filtered (5–2000 Hz), and digitized at a sampling rate of 10 kHz using the Map 1496 system (Nihon-Santeku Co., Osaka, Japan). During TMS measurement, each subject was seated comfortably in a reclining armchair.

The resting motor threshold (rMT) for the right APB muscle was defined as the minimal stimulator intensity sufficient to elicit five motor evoked potential (MEP) of >50 μV in a series of 10 stimuli delivered with at least 5 s intervals. To assess corticospinal excitability, we measured the peak-to-peak MEP amplitudes of both right APB and ADM muscles for 10 trials. The intensity of the test stimulus was adjusted to produce an MEP of ~1 mV from the target APB muscle before the intervention (SI 1 mV).

We measured short-latency intracortical inhibition and facilitation (SICI and ICF) to evaluate the cortical inhibitory and excitatory neural circuits. Paired-pulse magnetic stimuli were applied over the left M1, with a subthreshold conditioning stimulus (SC) at 80% of the rMT followed by a suprathreshold test stimulus (TS) at SI 1 mV with interstimulus intervals (ISIs) of 3 and 12 ms, respectively (Rossini et al., 2015). The test MEP amplitudes were adjusted to be constant at ~1 mV throughout the experiment. The size of the mean conditioned response for SICI and ICF (10 trials each) was expressed as a percentage of the size of the mean test response alone. These techniques allowed us to investigate the different pools of cortical interneurons that modulate the inhibitory and facilitatory neural circuits (Paulus et al., 2008; Badawy et al., 2012).

### Experimental procedures

**Figure 1:**
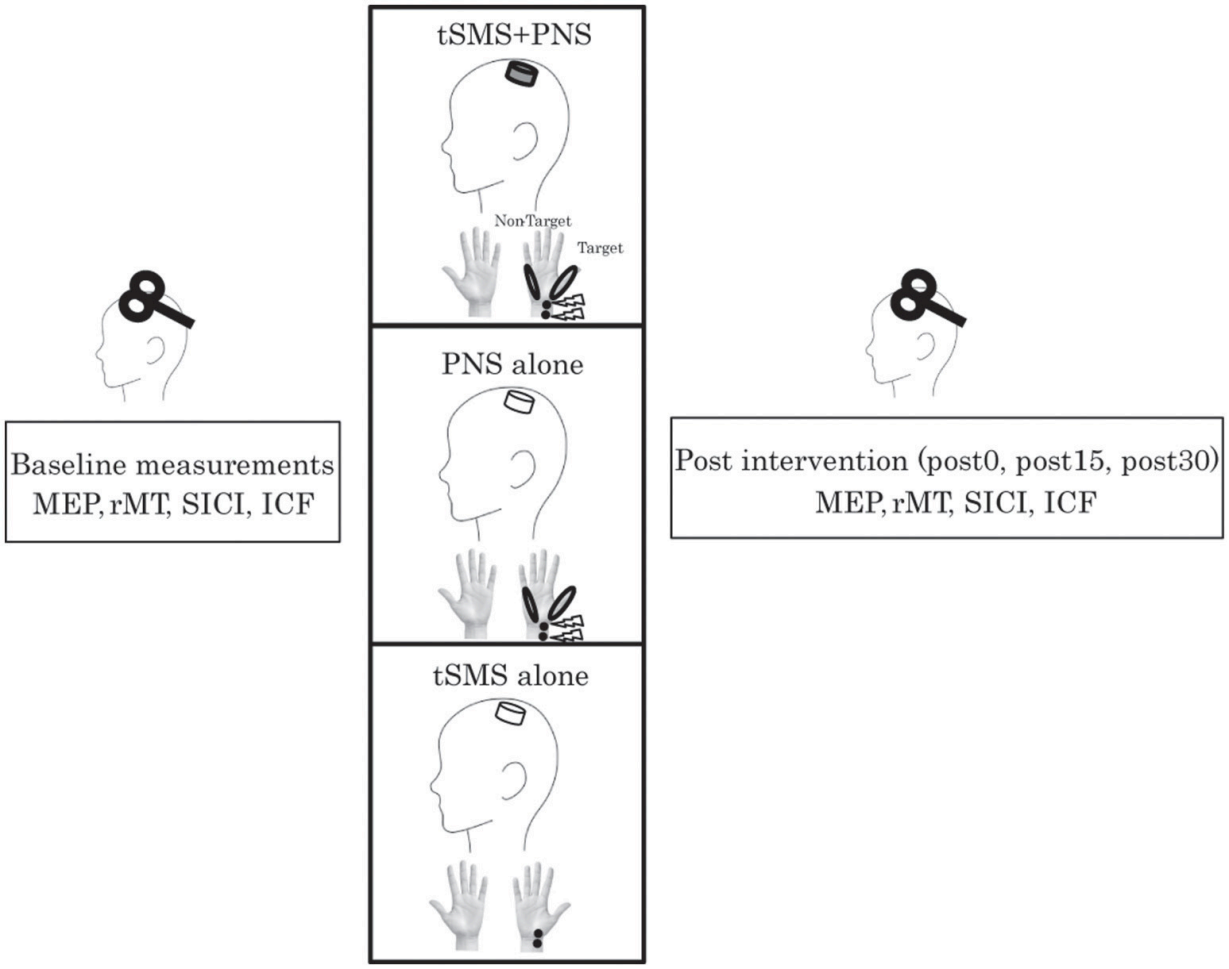
**Healthy volunteers received peripheral nerve stimulation on right median nerve combined with transcranial statistic magnetic stimulation (tSMS) over left M1 or sham stimulation**. We recorded the motor evoked potential (MEP) from the right abductor pollicis brevis (APB) and abductor digiti minimi (ADM) muscles to assess change in corticospinal and intracortical excitability immediately before and after 5 min of intervention. In each block, we assessed the resting motor threshold (rMT), short-latency intracortical inhibition (SICI) at an interstimulus interval (ISI) of 3 ms and intracortical facilitation (ICF) at an ISI of 12 ms. Evaluation was done just before, immediately, 15 min, and 30 min after intervention.

Subjects were asked to lie on a reclining chair to apply tSMS using a compact neodymium magnet and nonmagnet as sham stimulation. They were randomly assigned to two equal-sized groups (real and sham single-blind) (Figure 1). Subjects were asked if they received real (tSMS+PNS) or sham (Sham+PNS), and we confirmed that they were not aware of it. Each subject, therefore, underwent either real or sham stimulation in combination with PNS of the right hand. The peripheral and cortical stimulations were applied at the same time. We have set intervention for 5 min in order to investigate the effect of co-stimulation. In addition to these experiments, we executed the control experiment in order to confirm the dependence of tSMS-induced effects on its duration (tSMS alone). We tested the effects of 5 min of tSMS with sham PNS.

Regarding the cortical excitability changes induced by tSMS, we measured the MEP amplitudes and rMT. In addition to these parameters, we measured the SICI/ICF for the right APB before, 0, 15, 30 min after intervention.

### Data analysis

Although the present experiment is not designed as a double-blind study, for MEP measurement all the data were stored in a computer, and a blinded researcher checked the data without knowing the experimental information. The normal distribution was tested using the Kolmogorov–Sminov test.

First, two-way repeated-measures analysis of variance (ANOVA) was conducted to analyze the effects of three interventions on cortical excitability. The effect of interventions and time course on TMS parameters (MEP, rMT, SICI, and ICF) was examined with Group (tSMS+PNS, sham+PNS, and tSMS alone) and Time (pre, post-0, post-15, post-30). In the case of significant interaction effects, the Bonferroni correction for multiple comparisons was used as *post-hoc* analyses in order to compare with pre condition. All statistical analyses were performed using SPSS (IBM, Armonk, NY, USA), and alpha level was set at *p* < 0.05 for all tests. All data are given as the mean SEM. In the case of significant interaction effects, the Bonferroni correction for multiple comparisons was used as *post-hoc* analyses in order to compare with pre condition. All statistical analyses were performed using SPSS (IBM, Armonk, NY, USA), and alpha level was set at *p* < 0.05 for all tests. All data are given as the mean SEM.

## Results

To delineate the physiological mechanism of the effect of the combination tSMS with PNS, detailed TMS measurements were performed.

**Figure 2:**
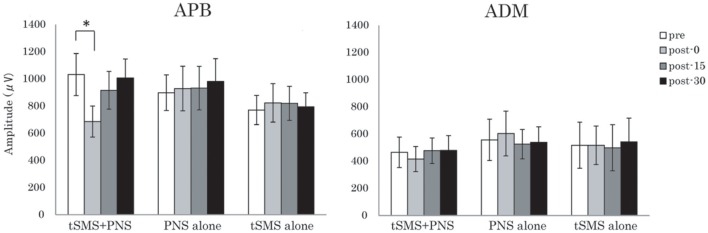
**Effects of the combination of tSMS and PNS on the MEP amplitude measured in the right APB and ADM muscles**. The mean MEP amplitude in APB muscle was significantly decreased immediately after the intervention combining tSMS and PNS but not after the other sham condition. ^*^p < 0.05 between pre and post-0. Error bars are standard errors of the mean.

Regarding the MEP amplitude for the right APB, two-way repeated-measures ANOVA showed no significant main effect of Time, but significant interaction of Group × Time [*F*_(3, 39)_ = 2.20 (*p* = 0.048)]. *Post-hoc* analysis revealed a significant suppression of MEP amplitude in post-0 (*p* < 0.011) of the tSMS+PNS group compared with pre condition, suggesting that tSMS combined with PNS influenced MEP amplitude immediately after intervention (Figure 2). By contrast, there was no significant effect of Time and Time × Group in the right ADM. A summary of the mean amplitude of both muscles is given in Table 1.

**Table 1:** **Changes of the time-course in TMS parameters for the right abductor pollicis brevis muscle**.

	**MEP(APB)**				**MEP(ADM)**			
	**Pre**	**Post-0**	**Post-15**	**Post-30**	**Pre**	**Post-0**	**Post-15**	**Post-30**
tSMS+PNS	1032.1±154.7	686.1 ± 113.9	916.0 ± 139.1	1006.9 ± 139.7	465.6 ± 112.1	415.8 ± 92.8	477.2±94.1	479.2 ± 109.3
PNS alone	898.2±131.4	929.1 ± 163.7	932.3 ± 160.3	980.7 ± 168.7	557.4 ± 152.0	603.9 ± 164.8	525.8±108.1	539.5 ± 113.4
tSMS alone	770.8±107.3	823.4 ± 141.6	820.3 ± 125.0	793.9 ± 104.0	517.6 ± 169.8	517.6 ± 141.6	498.9±169.9	542.9 ± 174.4
**rMT(APB)**								
	**Pre**	**Post-0**	**Post-15**	**Post-30**				
tSMS+PNS	54.3±2.6	55.8 ± 2.4	54.7 ± 2.7	54.5 ± 3.0				
PNS alone	52.6±1.6	52.9 ± 1.5	52.8 ± 1.5	52.2 ± 1.5				
tSMS alone	57.8±2.1	57.9 ± 2.2	58.0 ± 2.1	57.3 ± 2.0				
	**SICI(APB)**				**SICI(ADM)**			
	**Pre**	**Post-0**	**Post-15**	**Post-30**	**Pre**	**Post-0**	**Post-15**	**Post-30**
tSMS+PNs	0.602±0.081	0.456 ± 0.064	0.529 ± 0.040	0.545 ± 0.088	0.476 ± 0.082	0.467 ± 0.058	0.504±0.106	0.513 ± 0.081
PNS alone	0.575±0.088	0.548 ± 0.066	0.593 ± 0.070	0.598 ± 0.070	0.572 ± 0.083	0.580 ± 0.094	0.576±0.089	0.583 ± 0.113
tSMS alone	0.507±0.081	0.539 ± 0.071	0.517 ± 0.092	0.526 ± 0.073	0.615 ± 0.067	0.597 ± 0.076	0.640±0.093	0.621 ± 0.086

Values are mean ± SED. MEP, motor evoked potential; rMT, rest motor threshold; SICI, short-term intracortical inhibition; APB, abductor pollicis brevis; ADM, abductor digiti minimi; tSMS, transcranial static magnetic stimulation; PNS, peripheral nerve stimulation.

**Figure 3:**
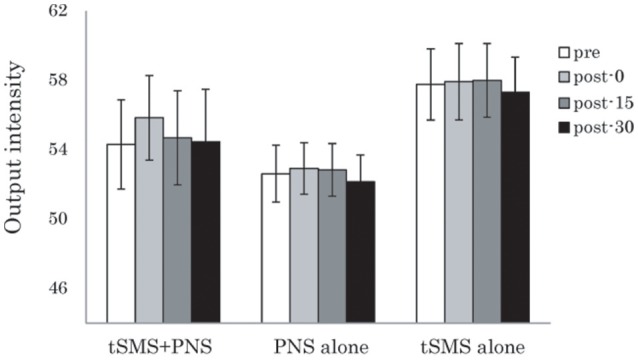
**Effects of the combination of tSMS and PNS on the rMT measured in the right APB muscle**. There were no significant changes of right APB. Error bars are standard errors of the mean.

For rMT, although two-way repeated-measures ANOVA showed no significant effects of Time and Group interaction, there was the tendency of increase immediately after tSMS+PNS intervention (Figure 3).

**Figure 4:**
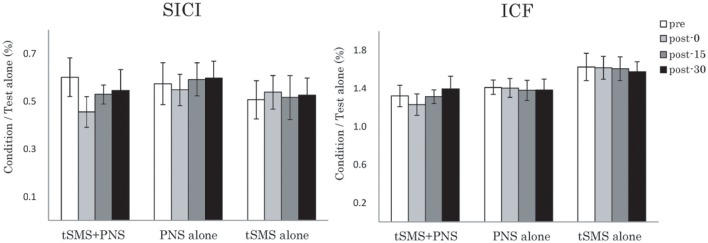
**Effects of combining tSMS with PNS on the SICI and ICF in the right APB muscle**. There were no significant changes of right APB. Error bars are standard errors of the mean.

In the cortical inhibitory and excitatory neural circuit, although two-way repeated-measures ANOVA showed no significant effects of Time and Time × Group interactions for both muscles, it also showed the trend toward of enhancement of SICI immediately after intervention of tSMS+PNS (Figure 4).

## Discussion

We found that tSMS to M1 combined with PNS to the median nerve produced a focal reduction in mean MEP amplitudes in the APB but not the ADM. We also confirmed that the lack of suppressive effects of APB in tSMS alone with short duration is in accord with the previous observation (Oliviero et al., 2011). Moreover, sham tSMS with PNS failed to induce any change. These findings suggested that the synaptic activation induced by a combination of tSMS with PNS can lead to somatotopically focal modulation in the cortical function. Our results suggested that the combination of tSMS and PNS can induce the somatotopic focal excitability change in cortical motor area.

This study revealed a significant decrease in MEP amplitude of the right APB only after the tSMS with PNS. However, this change of cortical excitability did not generalize to the right ADM muscle, which was an adjacent muscle innervated by a different nerve. This result suggested that an interesting feature of the combination of tSMS and PNS is the somatotopic focal effects which may help to shape the regional pattern of reorganization. This somatotopy is consist of other combining stimulation protocols (Stefan et al., 2000; Wolters et al., 2003; Koganemaru et al., 2009; Rizzo et al., 2009).

Our prediction was that concurrent PNS will potentiate the plastic change if the stimulation is given with tSMS. Consistent with our hypothesis, paired stimuli were tended to induce reduction in resting excitability of corticospinal output neurons. In contrast, sham tSMS with PNS for 5 min failed to induce significant change in the MEP amplitude. Previous PNS studies have demonstrated that more than 10 min of repeated PNS were required to provoke consistent increases in corticospinal excitability (Ridding et al., 2000, 2001; Pyndt and Ridding, 2004; Quartarone et al., 2006). On the other hand, original study (Oliviero et al., 2011) has reported that tSMS exposure less than for 10 min failed to reduce the MEP amplitude after the end of stimulation. The results in the present study suggested that the duration of tSMS for 5 min can suppress the cortical excitability if PNS was simultaneously applied. Although Kirimoto reported the loss of suppression of SEPs when tSMS was simultaneously combined with SEPs recording (Kirimoto et al., 2014), the results cannot be directly comparable to the present results. Firstly, our study measured the corticospinal excitability which was not measured in the previous study. Moreover, the parameters of peripheral nerve stimulation were totally different (1 Hz vs. 3.3 Hz). Concurrent afferent stimulation of peripheral nerves may produce an enhanced reduction effect of the corticospinal output.

Regarding PNS, it has been reported that high-frequency PNS (90 Hz) applied over the hand muscles in healthy volunteers for 30 min was associated with a decrease of sensory threshold and parallel decrease of corticospinal excitability (Mima et al., 2004). It suggested that long-term intervention was needed to modulate corticospinal excitability by PNS alone, and the additional tSMS exposure may potentiate the suppression effect induced by PNS. By contrast, it has also reported that prolonged PNS (>120 min) could enhance excitability in the contralateral M1 (Ridding et al., 2000, 2001). The divergence of the PNS-induced cortical excitability change might be related to the different stimulus frequencies. In patients with chronic stroke, PNS has been shown to transiently improve motor performance (Sawaki et al., 2006; Celnik et al., 2007) although little is known about the mechanisms and ability of PNS to modulate the effects of motor training. Further studies would be necessary to test the underling neural mechanism of PNS-induced cortical excitability change.

One of the plausible explanations of the effect of combining NIBS with PNS may be related to Brain-derived neurotrophic factor (BDNF) secretion. A recent *ex vivo* animal study in mice, in which anodal DCS applied to M1 slices was coupled with low-frequency synaptic stimulation, showed to induce long-term synaptic plasticity (Fritsch et al., 2010). Notably, these effects required activity-dependent BDNF secretion, a finding that was in agreement with previous demonstrations of the role of BDNF in NIBS-induced plasticity (Cheeran et al., 2008; Antal et al., 2010). BDNF is crucial for human motor learning, thus modulation of BDNF by external stimulation may help control the neuroplastic potential. Since the BDNF mechanism associated with cortical excitability change is still speculative, further study would be needed.

In addition to the decrease of MEP amplitudes, we found the increase of rMT just after the tSMS in accordance with previous studies (Silbert et al., 2013; Nojima et al., 2015). Although the basis of rMT is not fully certain yet, pharmacological research has suggested that it is modulated by membrane excitability (Ziemann et al., 1996a,b). These suggested that intrinsic excitability could be modulated by tSMS and may underlie some of the post-stimulation effects on cortical excitability.

Our previous study showed that tSMS can enhance the GABAergic system (Nojima et al., 2015). SICI is measured in a paired-pulse TMS protocol involving a subthreshold conditioning stimulus followed by a suprathreshold test stimulus with a short interstimulus interval of 1–5 ms (Kujirai et al., 1993; Nakamura et al., 1997; Chen et al., 1998). This inhibitory effect is thought to result primarily from activation by the conditioning stimulus of low threshold GABAergic interneurons in the cortex (Ziemann et al., 1996c; Di Lazzaro et al., 2002; Paulus et al., 2008). Our results suggested that suppression of focal M1 function may be partly related to the modulation of the GABAergic system.

The effect of tSMS on cortical excitability has also been confirmed in the other brain areas (Kirimoto et al., 2014; Aguila et al., 2016), and was reported that had created a reversible cortical scotoma in the animal experiment. Several animal studies reported that tSMS interfere with neural function (Rosen and Lubowsky, 1987; McLean et al., 2003, 2008; Coots et al., 2004; Aguila et al., 2016). Of these, it suggested that tSMS directly interfere with the functioning of membrane ion channels and consequently with the generation of action potentials (Coots et al., 2004), possibly due to the diamagnetic anisotropic properties of membrane phospholipids (Rosen, 2003b; Miyakoshi, 2005). Previous studies revealed that the activation kinetics of both sodium (Rosen, 2003a; Coots et al., 2004) and calcium (Rosen, 1996) channels were transiently affected during tSMS. The hypothesis is that tSMS would cause reorientation of membrane phospholipids, which would cause a deformation of ion channels embedded in the membrane and therefore altering their activation kinetics (Rosen, 2003b). Other possible influences have been postulated effects on cellular growth and size alterations of the cell cytoskeleton (Rosen and Chastney, 2009).

Regarding the strength of the magnetic field, recent study reported that magnet used in this study was in range between 120 and 200 mT 2–3 cm from the surface of the magnet (Rivadulla et al., 2014; Kirimoto et al., 2016). Therefore, it seems that this range is enough to obtain biological effects.

On the other hand, the magnet size we used in the present study was slightly bigger than the previous study. In the control experiment, we confirmed that tSMS for 5 min using our magnet does not change the M1 excitability, which is consistent with the previous study (Oliviero et al., 2011). However, since the effect of magnet size/strength and duration on M1 excitability has not been systematically investigated, further studies would be necessary to clarify these points.

Previous studies have already suggested that the effect of tSMS on excitability change was disappeared for couple min after removing the magnet (Roshan et al., 2003; Ortu et al., 2008). Due to the limitation of the time during the experiment, we tested only for the hot spot for the APB muscle. It is possible that the small test MEP amplitude in ADM might influence on the results of this study. It was reported that changes in the amplitude of the test MEP had markedly different effects on SICI, especially only slight inhibition in weaker test MEP amplitude (Ziemann et al., 1996a; Sanger et al., 2001). However, the amount of SICI in the ADM (0.572 ± 0.083) at the baseline was similar to that in the APB. It suggests that the SICI phenomenon in both muscles would have been occurred in a proper way.

This study provides a new combined tSMS protocol that can be used for the induction of somatotopically focal M1 excitability change. Moreover, we confirmed that 5 min of co-stimulation induced a decrease in the excitability of the corticospinal output from the stimulated M1. These results suggested that tSMS exposure could be a valuable tool in research studies of cortical function. And enhancement of SICI function in somatotopically focal brain area by combining with PNS might be a new promising therapeutic tool for neurological disorders associated with GABA dysfunction, such as epilepsy (McLean et al., 2003, 2008) and dystonia (Ikoma et al., 1996; Garibotto et al., 2011; Boecker, 2013). Because somatotopic specificity is an important characteristic of this co-stimulation, we believe this protocol is suitable for a clinical therapeutic approach.

## Ethics statement

The Ethics Committee of Kyoto University Graduate School of Medicine (Kyoto, Japan) and Nagoya University Graduate School of Medicine (Nagoya, Japan). Each participant gave written informed consent before participation.

## Author contributions

IN and TM designed the study. IN performed the experiment and analyzed the data. IN, SK, TM interpreted results of the experiment. IN drafted the manuscript. IN and TM edited and revised the manuscript. All authors approved the final version of the manuscript.

## Disclosure

None of the authors have potential conflicts of interest to be disclosed. We confirm that we have read the Journal's position on issues involved in ethical publication.

### Conflict of interest statement

The authors declare that the research was conducted in the absence of any commercial or financial relationships that could be construed as a potential conflict of interest.
